# Analysis of Related Factors of Tumor Recurrence or Progression After Transnasal Sphenoidal Surgical Treatment of Large and Giant Pituitary Adenomas and Establish a Nomogram to Predict Tumor Prognosis

**DOI:** 10.3389/fendo.2021.793337

**Published:** 2021-12-14

**Authors:** Yike Chen, Feng Cai, Jing Cao, Feng Gao, Yao Lv, Yajuan Tang, Anke Zhang, Wei Yan, Yongjie Wang, Xinben Hu, Sheng Chen, Xiao Dong, Jianmin Zhang, Qun Wu

**Affiliations:** ^1^ Department of Neurosurgery, Second Affiliated Hospital, School of Medicine, Zhejiang University, Hangzhou, China; ^2^ Department of Statistical Office, The Affiliated Changsha Central Hospital, Hengyang Medical School, University of South China, Changsha, China; ^3^ Department of Neurosurgery, The Affiliated People’s Hospital of Ningbo University, Ningbo, China; ^4^ Department of Neurosurgery, The Affiliated Quzhou People’s Hospital of Wenzhou University, Quzhou, China

**Keywords:** pituitary adenoma, tumor recurrence, transnasal sphenoidal surgery, Cox regression model, nomogram

## Abstract

**Background:**

Pituitary adenoma (PA) is a benign neuroendocrine tumor caused by adenohypophysial cells, and accounts for 10%-20% of all primary intracranial tumors. The surgical outcomes and prognosis of giant pituitary adenomas measuring ≥3 cm in diameter differ significantly due to the influence of multiple factors such as tumor morphology, invasion site, pathological characteristics and so on. The aim of this study was to explore the risk factors related to the recurrence or progression of giant and large PAs after transnasal sphenoidal surgery, and develop a predictive model for tumor prognosis.

**Methods:**

The clinical and follow-up data of 172 patients with large or giant PA who underwent sphenoidal surgery at the Second Affiliated Hospital of Zhejiang University School of Medicine from January 2011 to December 2017 were retrospectively analyzed. The basic clinical information (age, gender, past medical history etc.), imaging features (tumor size, invasion characteristics, extent of resection etc.), and histopathological characteristics (pathological results, Ki-67, P53 etc.) were retrieved. SPSS 21.0 software was used for statistical analysis, and the R software was used to establish the predictive nomogram.

**Results:**

Seventy out of the 172 examined cases (40.7%) had tumor recurrence or progression. The overall progress free survival (PFS) rates of the patients at 1, 3 and 5 years after surgery were 90.70%, 79.65% and 59.30% respectively. Log-rank test indicated that BMI (P < 0.001), Knosp classification (P < 0.001), extent of resection (P < 0.001), Ki-67 (P < 0.001), sphenoidal sinus invasion (P = 0.001), Hardy classification (P = 0.003) and smoking history (P = 0.018) were significantly associated with post-surgery recurrence or progression. Cox regression analysis further indicated that smoking history, BMI ≥25 kg/m^2^, Knosp classification grade 4, partial resection and ≥3% Ki-67 positive rate were independent risk factors of tumor recurrence or progression (P < 0.05). In addition, the nomogram and ROC curve based on the above results indicated significant clinical value.

**Conclusion:**

The postoperative recurrence or progression of large and giant PAs is related to multiple factors and a prognostic nomogram based on BMI (≥25 kg/m^2^), Knosp classification (grade 4), extent of resection (partial resection) and Ki-67 (≥3%) can predict the recurrence or progression of large and giant PAs after transnasal sphenoidal surgery.

## Introduction

Pituitary adenoma (PA) is a benign neuroendocrine tumor that originates from adenohypophysial cells, and accounts for 10%-20% of all primary intracranial tumors (1, 2). The global prevalence of PA ranges from 80 to 100 cases per 100,000 (3). Pituitary adenomas disrupt the endocrine function of the pituitary gland, and the growing tumor mass can lead to headaches, vision problems and visual field changes. Giant PA is defined as tumors with the largest diameter ≥4 cm (4–6), and some investigators have defined tumors with the largest diameter ≥3 cm as large PA (5, 7, 8). Large and giant PAs tend to invade the region surrounding the saddle, starting from the bone of saddle bottom and then progressing to the sphenoid sinus. Tumor growth through the diaphragm sella compresses the optic chiasm and third ventricle, and involvement of the cavernous sinus leads to encasement of the internal carotid artery. Therefore, the internal carotid artery and peripheral nerves can be easily damaged during the operation. In addition, some lesions are tough in texture or accompanied by spontaneous stroke, which further increases the difficulty of surgery and increases the risk of recurrence or progression.

Surgical removal is the first-line treatment for most large and giant PAs, except for prolactinoma (9, 10). Since craniotomy causes greater damage to normal brain tissues and results in more postoperative complications, it is now gradually being replaced with the transnasal sphenoidal approach (11). Jankowski et al. were the first to successfully perform neuroendoscopy-guided transsphenoidal resection of PA (12). Nevertheless, regardless of whether the surgery is performed using neuroendoscopy or microscopy, or *via* the transnasal sphenoidal or transcranial approach, the surgical outcomes are still not satisfactory. Over 50%-72% of the cases have postoperative residual (13–16), and the recurrence rates at 5- and 10 years after surgery are 40% and 50% respectively (17). Even with gross total resection, at least 10-20% of the patients relapse within 5 to 10 years (2, 14), which greatly affects their quality of life.

Apart from postoperative residual, the recurrence of PA also depends on the tumor size and cavernous sinus invasion, which are risk factors for poor prognosis (14, 18, 19). In addition, the pathological subtype is also a factor influencing tumor recurrence and progression (20). It was reported that sparsely granulated GH adenomas and prolactin PAs in male patients are highly aggressive and have poor prognosis (21, 22).

Recurrence and progression of residual PA lesions impair the function of the pituitary gland. Therefore, long-term hormone replacement therapy is often required after surgery, which greatly reduces the quality of life of patients. In addition, surgical resection of the recurrent or evolved tumors increases the risk of vascular rupture, nerve injury and other adverse complications, which further worsens patient prognosis. In this study, we retrospectively analyzed the clinical, pathological, surgical and imaging data of 172 patients with large or giant PA to identify the risk factors of tumor recurrence and progression. In addition, we developed a nomogram to predict tumor recurrence or progression and assess postoperative prognosis.

## Materials and Methods

### Study Population

After obtaining institutional review board approval, the clinical data of 172 patients with large (>3 cm) or giant (>4 cm) PA that underwent surgery at The Second Affiliated Hospital Zhejiang University School of Medicine between January 2011 and December 2017 were retrospectively analyzed. The inclusion criteria were as follows: 1) histologically confirmed PAs with maximal diameter >3–4 cm (large) or >4 cm (giant), 2) underwent tumor resection through transnasal sphenoidal surgery, 3) no radiotherapy during the period of treatment, and 4) regular follow-up for a minimum of 6 months. Patients with serious intraoperative complications (such as internal carotid artery rupture or anesthesia accident), severe cardiopulmonary insufficiency, organ failure or other serious underlying diseases, anatomical variation or complicated with vascular disease (such as aneurysm), and those who underwent craniotomy for PA were excluded. The patients were re-examined 3-, 6- and 12 months after surgery, and yearly thereafter. Images of the pituitary were assessed by a neurosurgeon and a neuroradiologist during the follow-up period. Recurrence or progression was defined as significant enlargement (>2 mm in any direction) of the tumor remnants or the appearance of new masses detected by MRI during the follow-up (14).

### Data Collection

The patient data was divided into basic, radiological and pathological categories. Basic information included gender, age, history of hypertension, diabetes, smoking [patients who have smoked more than 100 cigarettes in lifetime were defined as having smoking history (23)], drinking, body mass index (overweight≥25kg/m^2^, not overweight<25kg/m^2^), function and operation methods (microscopy or neuroendoscopy). Radiological characteristics included cavernous sinus invasion, sphenoid sinus invasion, sella invasion, Hardy classification, Knosp classification, extent of resection [gross total resection (GTR), the extent of resection is greater than 95%; near total resection, NTR, the extent of resection is between 90% and 95%; subtotal resection, STR, the extent of resection is between 70%-90%; partial resection, PR, the extent of resection is less than 70% (8)]. The pathological classification, and P53 and Ki-67 positive rates were also collected.

### Data Analysis

Differences between subgroups were analyzed using Student’s t-test for the measurement data and the X^2^ test was used for categorical variables. The Kaplan-Meier survival curves were plotted and compared by log-rank tests using SPSS 21.0. Multivariate Cox regression analysis was performed to assess the independent predictive risk factors for tumor relapse, and a nomogram model was established with the R software version 4.0.3 (https://www.r-project.org, R package “rms”, “survival”.) to predict outcomes at the 1-, 3-, and 5-year follow-up. The performance of the nomogram was determined with ROC curve and calibration curve. Two-sided P values below 0.05 were considered statistically significant.

## Results

### General Characteristics

A total of 172 patients (83 females and 89 males) with pathologically confirmed PA were included. The mean age was 53.7 ± 12.7 years (range, 18-83 years), of which 109 patients (63.4%) were younger than 60 years and 63 patients (36.6%) were older than 60 years. Non-functional PA was detected in 150 patients (87.2%) and 22 patients (12.8%) had functional PAs. In addition, 101 patients (58.7%) had a BMI<25 kg/m^2^ and 71 patients (41.3%) had BMI≥25 kg/m^2^, 53 patients (30.8%) had a history of smoking, 60 patients (34.9%) had a history of alcohol consumption, 51 patients (29.7%) had a history of hypertension, and 17 patients (9.9%) had a history of diabetes. Based on the preoperative results of MRI and intraoperative observation, there were 150 cases of cavernous sinus invasion, 65 of sphenoid sinus invasion, and 152 cases of sella invasion. Due to the low number of patients in each group, the subgroups made on the basis of Knosp and Hardy classifications were pooled into several larger groups. For instance, the patients were divided into Knosp classification 0-1 (15 patients, 8.7%), 2-3 (92 patients, 53.5%) and 4 (65 patients, 37.8%), Hardy classification 1-2 (61 patients, 8.7%), 3 (77 patients, 55.3%) and 4-5 (34 patients, 37.8%). All patients underwent transnasal sphenoidal surgery, of which 112 patients (65.1%) were treated with neuroendoscopy and 60 (34.9%) with microscopy. GTR was performed in 28 cases (16.3%), NTR in 62 cases (36%), STR in 54 cases (31.4%) and PR in 28 cases (16.3%). According to the 2017 WHO classification of PA (some patients were not classified according to this standard, so this part of the results could not be included in the study), there was 1 case of somatotroph adenoma, 1 of lactotroph adenoma, 5 corticotroph adenoma (sparsely granulated corticotroph adenoma in 2 cases), 4 gonadotroph adenoma, 5 null cell adenoma, and 3 plurihormonal adenoma. Furthermore, 47 cases (27.3%) were P53 positive and 44 cases (25.6%) were Ki-67≥3% (**Table 1**).

**Table 1:** General characteristics.

Clinical Features	Groups	N of Patients (%)
Age	<60	109(63.4%)
	≥60	63(36.6%)
Clinical status	Non-functional PA	150(87.2%)
	Functional PA	22(12.8%)
BMI	<25 kg/m^2^	101(58.7%)
	≥25 kg/m^2^	71(41.3%)
Dietary Habit	Smoking	53(30.8%)
	Drinking	60(34.9%)
Past Medical History	Diabetes	17(9.9%)
	Hypertension	51(29.7%)
Hardy Classification	Grade 1~2	61(35.4%)
	Grade 3	77(44.8%)
	Grade 4~5	34(19.8%)
Knosp Classification	Grade 0~1	15(8.7%)
	Grade 2-3	92(53.5%)
	Grade 4	65(37.8%)
Structure of Tumor Invasion	Cavernous Sinus	150(87.2%)
	Sphenoid Sinus	65(37.7%)
	Sella	152(88.3%)
Operation Methods	Microscope	60(34.9%)
	Endoscope	112(65.1%)
Extent of resection	GTR	28(16.3%)
	NTR	62(36.0%)
	STR	54(41.4%)
	PR	28(16.3%)
Pathologic Results	P53+	47(27.3%)
	Ki-67≥3%	44(25.6%)

### Tumor Recurrence and Progression

**Figure 1:**
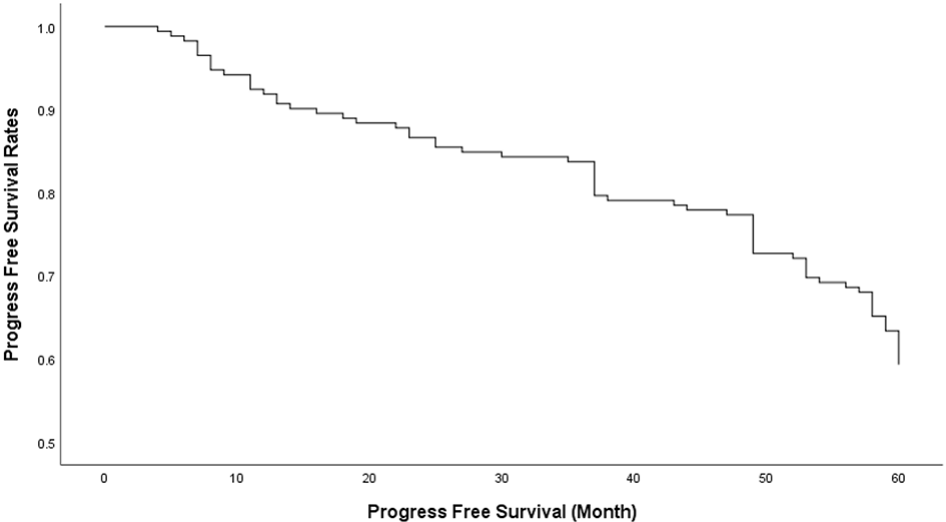
The overall progress free survival (PFS) rates of 1, 3 and 5 years after transnasal sphenoidal surgery was 90.70%, 79.65% and 59.30% respectively.

Seventy patients (40.7%) had recurrence or progression, and the overall progress free survival (PFS) rates 1-, 3-, and 5 years after surgery was 90.70%, 79.65% and 59.30% respectively (**Figure 1**).

### Univariate Analysis of Potential Risk Factors

The following factors were subjected to univariate analysis to identify those significantly associated with postoperative recurrence or progression of large and giant PAs: age, gender, history of hypertension and diabetes, smoking history, drinking history, BMI (<25 kg/m^2^/≧25 kg/m^2^), clinical function, surgical method (endoscopic/microscope), cavernous sinus invasion, spinous sinus invasion, suprasellar invasion, Hardy classification, Knosp classification, extent of resection (GTR >95%, NTR 90%-95%, STR 70%-90% and PR <70%), P53 and Ki-67 (<3%/≥3%). BMI (P <0.001), Knosp classification (P <0.001), extent of resection (P <0.001), Ki-67 (P <0.001), sphenoid sinus invasion (P =0.001), Hardy classification (P =0.003) and smoking history (P =0.018) were the significant risk factors of tumor recurrence or progression (**Table 2**).

**Table 2:** Univariate analysis results and mean PFS for each group.

Clinical Features	Groups	N	PFS	95%CI	X^2^	*P*
Age						
	<60	109	49.32	(46.04,52.61)	0.016	0.899
	≥60	63	51.30	(47.14,55.46)		
Gender						
	Female	78	56.73	(52.20,61.26)	3.413	0.065
	Male	94	50.59	(46.82,54.35)		
Hypertension						
	N	121	48.98	(45.67,52.30)	0.170	0.68
	Y	51	52.57	(48.90,56.24)		
Diabetes						
	N	155	49.57	(46.75,52.39)	0.414	0.52
	Y	17	54.41	(50.13,58.69)		
Smoking						
	N	119	54.35	(52.30,56.40)	5.588	0.018
	Y	53	40.38	(34.11,46.65)		
Drinking						
	N	112	49.30	(46.09,52.5)	2.889	0.089
	Y	60	51.45	(47.1,55.81)		
BMI						
	<25 kg/m^2^	101	53.60	(50.88,56.33)	13.708	<0.001
	≧25 kg/m^2^	71	44.99	(40.31,49.66)		
Clinical status						
	N	150	55.35	47.64,53.06	0.282	0.596
	Y	22	57.96	39.85,56.06		
Operation Methods						
	Endoscope	112	50.50	(47.43,53.57)	0.072	0.788
	Microscope	60	49.20	(44.53,53.88)		
Cavernous Sinus Invasion						
	N	22	47.32	(39.58,55.06)	0.924	0.336
	Y	150	50.45	(47.72,53.18)		
Sphenoid Sinus Invasion						
	N	107	52.95	(50.22,55.69)	10.816	0.001
	Y	65	45.26	(40.34,50.19)		
Sella Invasion						
	N	51	48.02	(43.39,52.65)	3.229	0.072
	Y	152	50.90	(47.80,54.00)		
Hardy Classification						
	Grade 1~2	61	53.26	(49.98,56.54)	11.579	0.003
	Grade 3	77	51.49	(47.72,55.27)		
	Grade 4~5	34	41.00	(33.83,48.17)		
Knosp Classification						
	Grade 0~1	15	59.07	(57.56,60.57)	24.018	<0.001
	Grade 2~3	92	52.71	(49.52,55.90)		
	Grade 4	65	44.20	(39.46,48.94)		
Extent of resection						
	GTR	28	57.82	(55.02,60.62)	37.748	<0.001
	NTR	62	52.73	(49.02,56.44)		
	STR	54	50.26	(45.50,55.02)		
	PR	28	35.93	(28.55,43.31)		
P53						
	Negative	125	51.41	(48.56,54.25)	1.561	0.211
	Positive	47	46.43	(40.89,51.96)		
Ki-67						
	<3%	128	53.97	(51.58,56.36)	39.916	<0.001
	≥3%	44	38.64	(32.44,44.83)		

**Figure 2:**
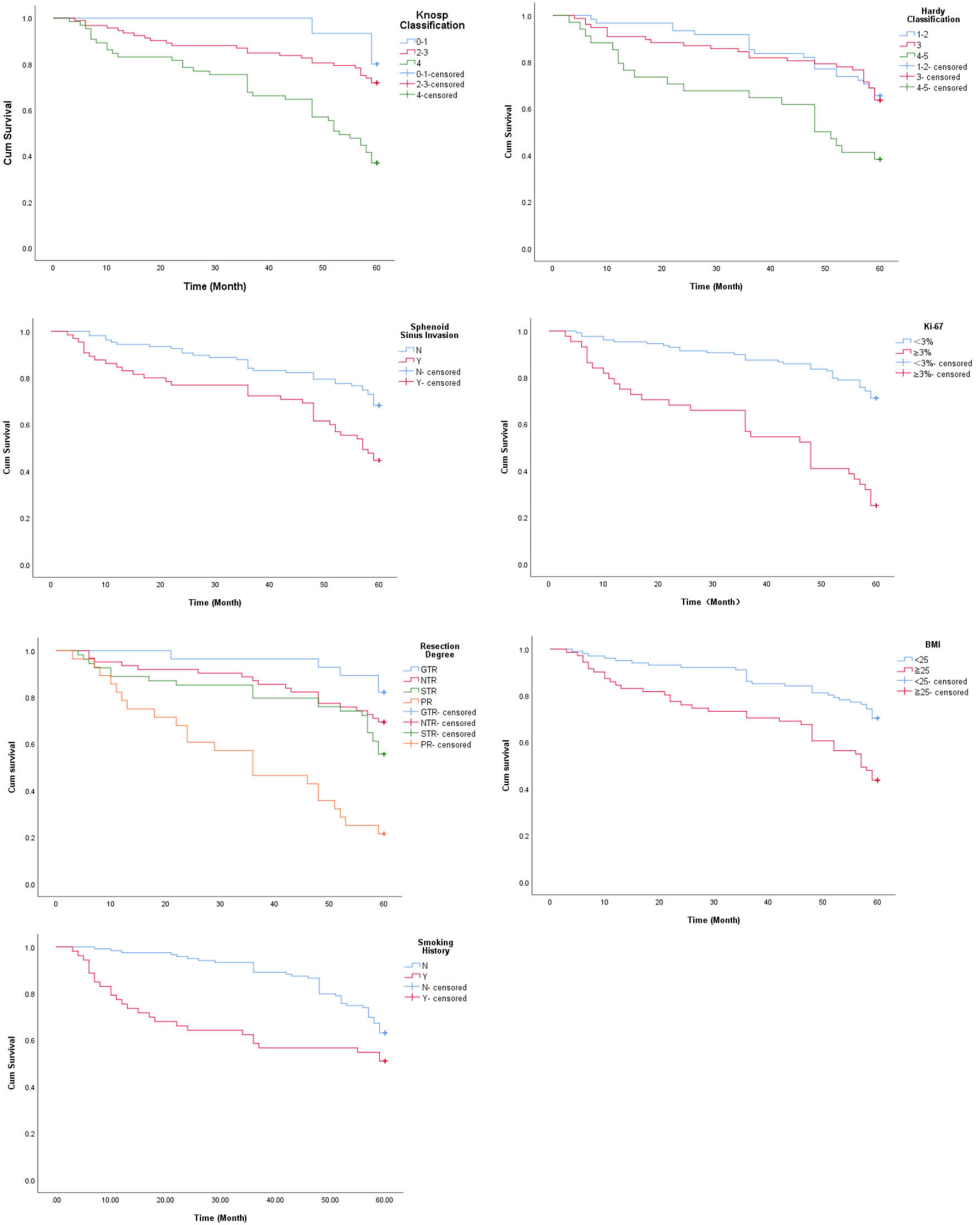
Survival curves of patients stratified on the basis of Knosp classification, Hardy classification, sphenoid sinus invasion, Ki-67, extent of resection, BMI and smoking history.

Studies show that tumor size and the extent of invasion into the sphenoid sinus significantly affect post-operative recurrence or progression of PA (14, 24, 25). The mean PFS duration (**Table 2**) and survival rate (**Figure 2**) of patients with Grade 4 Knosp classification were significantly less compared to that of the other groups (P < 0.001). Likewise, the mean PFS of Grade 4-5 Hardy classification was only 41 months, and the survival rate was significantly lower compared to that of grades 1-2 and 3 (P = 0.003). The mean PFS of patients with sphenoid sinus invasion was 45.26 months compared to 52.59 months in patients without sphenoid sinus invasion (P = 0.001), and the survival rates were significantly different (**Figure 2**). Ki-67 expression is an indicator of tumor cell proliferation and invasion (26). The mean PFS of patients with highly proliferative tumors (Ki-67 ≥3%) was only 38.64 months compared to the 53.97 months in patients with less active tumors (Ki-67 <3%) (P < 0.001). The extent of surgical resection was determined on the basis of surgical records and postoperative imaging results, and the patients were divided into GTR, NTR, STR and PR groups. The shortest mean PFS and survival rate was seen in the PR group (P < 0.001 versus all).

Although body mass index (BMI) and smoking history are not directly related to PA, univariate analysis suggested that both factors have a significant impact on postoperative recurrence or progression of large and giant PAs. Based on the pre-operative BMI, the patients were divided into overweight (BMI≧25 kg/m^2^) and non-overweight (BMI < 25 kg/m^2^) groups. The mean PFS of the overweight patients was 44.99 months as opposed to 53.6 in patients with healthy BMI (P< 0.001). Furthermore, patients with a history of smoking had a shorter mean PFS compared to the non-smokers (40.38 months vs 54.35 months). As shown in **Figure 2**, there was a significant difference in PFS between the two groups (P = 0.018).

### Independent Risk Factors for Postoperative Recurrence or Progression of Large and Giant Pas

The factors identified in the univariate analysis were incorporated into the multivariate Cox regression model. Smoking history (HR=3.103, 95% CI: 1.812-5.314), BMI (≧25 kg/m^2^) (HR=1.997, 95% CI:1.206-3.306), Knosp classification (grade 4) (HR=4.093, 95% CI:1.144-14.649), extent of resection (PR) (HR=3.723, 95% CI:1.152-12.033) and Ki-67 positivity (≥3%) (HR=4.639, 95% CI:2.686-8.013) were identified as the independent risk factors (**Table 3**).

**Table 3:** Multivariate analysis results.

Variables	B	SE	Wald	*P*	HR	95.0% CI for HR
Smoking History	1.132	0.274	17.017	<0.001	3.103	1.812-5.314
BMI(≧25 kg/m^2^)	0.691	0.257	7.222	0.007	1.997	1.206-3.306
Sphenoid Sinus Invasion	0.403	0.262	2.354	0.125	1.496	0.894-2.501
Hardy Classification						
Grade 1-2					1.000	
Grade 3	0.064	0.299	0.045	0.831	1.066	0.593-1.914
Grade 4-5	0.599	0.362	2.739	0.098	1.821	0.895-3.703
Knosp Classification						
Grade 0-1					1.000	
Grade 2-3	0.678	0.629	1.160	0.282	1.970	0.574-6.763
Grade 4	1.409	0.651	4.692	0.030	4.093	1.144-14.649
Extent of resection						
GTR					1.000	
NTR	0.503	0.520	0.936	0.333	1.653	0.597-4.579
STR	0.692	0.519	1.779	0.182	1.997	0.723-5.519
PT	1.315	0.598	4.825	0.028	3.723	1.152-12.033
P53 (-)	-0.259	0.298	0.754	0.385	0.772	0.430-1.385
Ki-67 (≥3%)	1.535	0.279	30.290	<0.001	4.639	2.686-8.013

### Establishment and Validation of Predictive Nomogram

**Figure 3:**
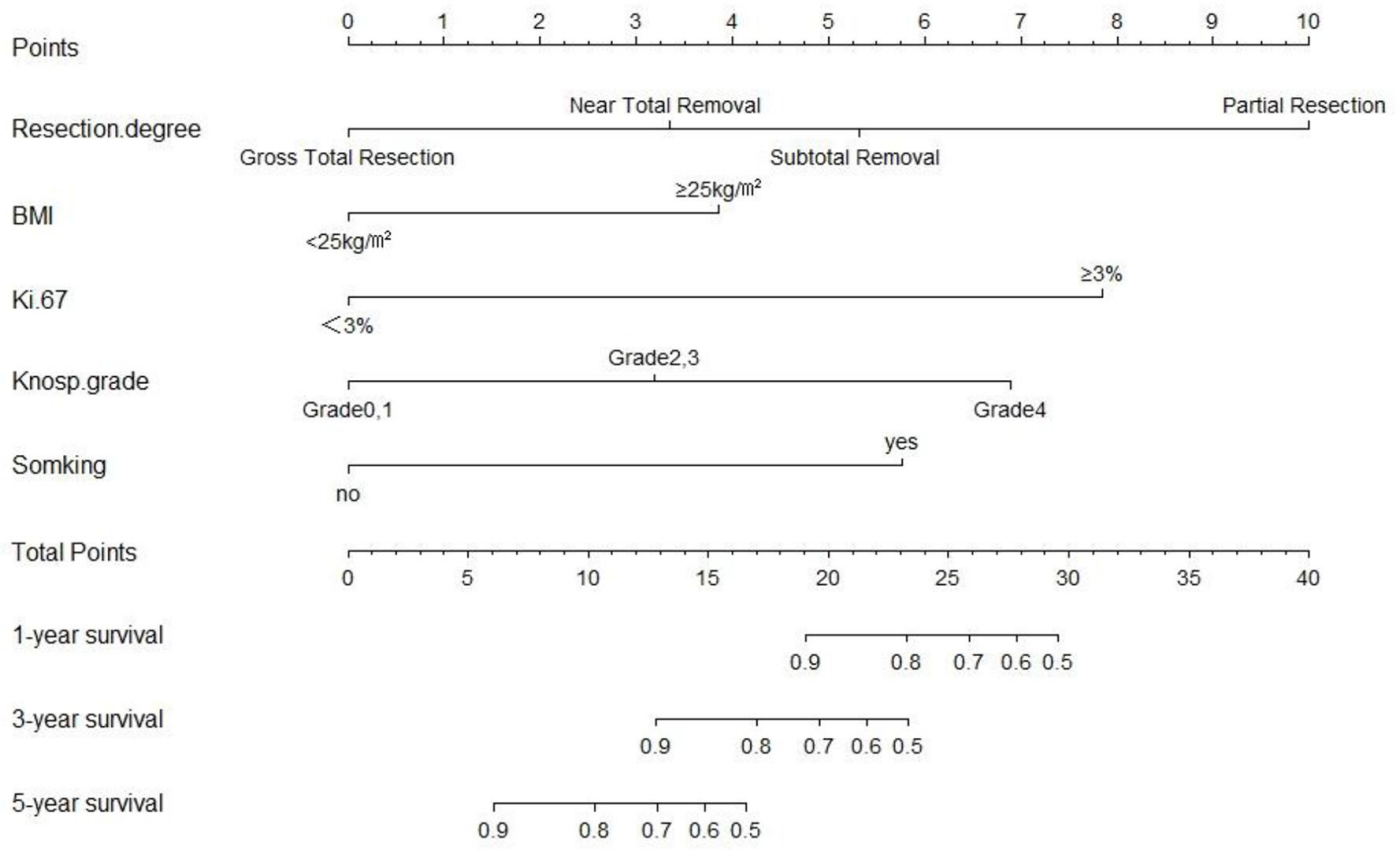
The predictive nomogram based on resection degree, BMI, Ki-67, Knosp classification and smoking history.

**Figure 4:**
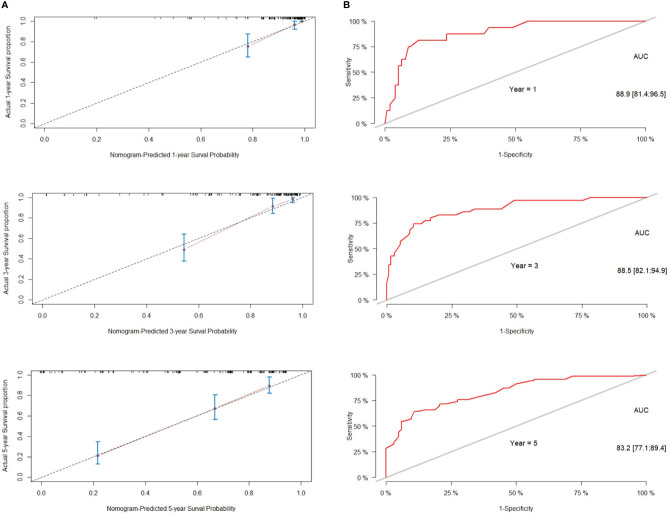
Performance of the nomogram for predicting tumor recurrence or progression. **(A)** calibration curve of the nomogram. **(B)** The predictive performance of the nomogram was assessed by receiver operator characteristics (ROC) analysis and area under the curve (AUC).

We next established a nomogram based on the extent of resection, BMI, Ki-67, Knosp classification and smoking to predict the 1-, 3-, and 5-year prognosis of patients (**Figure 3**). Each risk factor was designated a score, and the sum of the five scores in the individual patients corresponded to the probability of PFS 1, 3 and 5 years after surgery. The calibration curve and ROC were used to confirm the clinical value of this model, and indicated good agreement between the predicted and observed values (**Figure 4A**). As shown in **Figure 4B**, the area under the ROC curves (AUC) for 1-, 2-, and 3-year survival were 0.889, 0.885 and 0.832 respectively, indicating satisfactory accuracy.

## Discussion

In this study, we retrospectively analyzed the clinical data of 172 patients with large or giant PA, and identified multiple risk factors for postoperative recurrence or progression. A prognostic nomogram was established based on these factors, which could predict survival with satisfactory accuracy.

The extent of surgical resection is the main factor influencing tumor recurrence or progression. In this study, we defined GTR (>95%), NTR (90%-95%), STR (70%-90%) and PR (<70%) on the basis of postoperative imaging results and surgical records. PR was significantly associated with tumor recurrence or progression (HR: 3.302; *P*=0.048). The transsphenoidal approach with neuroendoscopy can improve the extent of surgical resection (19, 27). Fathalla et al. reviewed 65 patients with acromegaly, and found that the extent of resection was significantly greater in patients who underwent endoscopy as opposed to the microscopy group (61% vs 42%, P=0.05). Furthermore, endoscopy also improved the extent of resection (48% vs 14.2%, P=0.09) when the tumor invaded the cavernous sinus (27). In our cohort however, the mean PFS of patients who were treated with neuroendoscopy and microscopy were 50.50 and 49.2 months respectively, indicating that the surgical method did not have any impact on the postoperative recurrence and progression.

The invasiveness of PA is also a risk factor for postoperative recurrence or progression. Similar to the diaphragm sella, the wall of the cavernous sinus is a thin dural bag (28), and most patients in our cohort had cavernous sinus invasion (87.2%) and/or suprasellar invasion (88.4%). However, only sphenoid sinus invasion was identified as a risk factor of postoperative tumor recurrence or progression. This is consistent with the views of some researchers that sphenoid sinus invasion rather than cavernous sinus invasion is a more indicative of “invasiveness” (29, 30). In one case of a particularly aggressive tumor, the growing mass passed through the bone of sellar floor to invade the sphenoid sinus in a cathepsin K-dependent manner (31). The size and invasion of PA are graded by Knosp classification and Hardy classification that are based on preoperative imaging data, and are used by neurosurgeons in preoperative evaluation and prediction of surgical. We found that both were significantly associated with the recurrence or progression of large and giant PAs after surgery, and Knosp 4 was an independent risk factor, which is highly associated with tumor recurrence. Thus, Knosp classification may play an important role in predicting tumor recurrence as reported in previous studies (32–34).

The invasiveness of adenomas and other tumors is also evaluated in terms of pathological parameters, such as mitotic figures, Ki-67 index (35, 36) and p53 expression in the malignant tissues (26). Petry et al. conducted a retrospective analysis of 52 patients with PA and divided them into Ki-67 ≥3% and Ki-67 <3% groups, and found that high Ki-67 expression was correlated with more aggressive tumor growth and higher recurrence rate (67%vs 17%, P=0.03) (37). Consistent with this, we found that the mean PFS of patients with higher Ki-67 index tumors (≥3%) was significantly lower compared to those with < 3% Ki-67 positivity. Furthermore, Ki-67 ≥ 3% was identified an independent risk factor of postoperative recurrence or progression. However, P53 expression did not have any impact on the mean PFS. According to WHO 2017 classification, the pathological subtypes of PA include sparsely granulated somatotroph adenoma, lactotroph adenoma (in men), pluri-hormonal PIT-1 positive adenoma, silent corticotroph adenoma and crooke cell adenoma, all of which are considered high-risk (38). Due to limitations of specific markers and staining techniques, we were unable to include this pathological classification in our study. Therefore, predicting the proliferative potential of large and giant PAs classified on the basis of more rigorous pathological criteria remains to be elucidated.

The prognosis of PA is evaluated according to imaging features (tumor site, Knosp classification etc.) and pathological results (pathological classification, Ki-67, P53 etc.) (39), which do not take into account other clinical factors. In this study, we found that BMI ≧ 25 kg/m^2^ and a history of smoking were independent risk factors of postoperative recurrence or progression. Obesity is an established risk factor for intracranial tumors (40, 41), and promotes tumor development through chronic insulin resistance, hyperinsulinemia and enhanced IGF-1 activity. In fact, more than half of meningiomas overexpress IGF-1 receptors, and IGF-1 can promote the growth of meningioma cells *in vitro* (42). Furthermore, overexpression of IGF-1 receptors in glioma cells promotes their proliferation and inhibits apoptosis (43, 44). In this study, the mean PFS of overweight patients (BMI≥25 kg/m^2^) was significantly shorter than that of patients with a healthy weight (BMI < 25 kg/m^2^). All these evidences provide new insights into the molecular mechanisms underlying recurrence or progression of large and giant PAs after surgery. Several studies have shown that smoking is a high-risk factor for lung cancer, bladder cancer, head and neck cancer and other cancers (45–47). Taken together, the correlation of general health and smoking with PA progression warrants further study.

Although the association between PA recurrence or progression and clinical variables has been reported previously (48–50), multiple variables are rarely incorporated to assess prognosis. Nomograms are now widely used to predict the prognosis of other tumors, since they can simplify the statistical model, and estimate the probability of an event (such as death or recurrence) with a single value (51). In addition, a nomogram can integrate multiple prognostic variables and determinants that simulate complex biological and clinical scenarios for personalized medicine (52). On the basis of the univariate and multivariate models, we developed a nomogram to predict the recurrence or progression of PA after transnasal sphenoidal surgery by integrating the independent risk factors, and found that the model can predict tumor recurrence 1-, 3- and 5 years after surgery with satisfactory accuracy. This nomogram provided probability estimates that may be useful for individual patients, and help predict tumor recurrence or progression after surgery. We can use this scoring system after surgery by adding up the scores for all factors, objectively evaluate the possibility of tumor recurrence or progression, and provide a reference for clinical treatment to develop an individual follow-up plan.

However, this study has certain limitations that should be considered. First, this was a single-center retrospective study, which warrants further validation of the predictive model on a larger, multicenter cohort. Second, the association of the pathological subtypes of PA with tumor recurrence or progression should be further explored with a uniform and strict standard. In conclusion, partial resection, BMI ≧25 kg/m^2^, Ki-67 ≥3%, Knosp classification grade 4 and smoking increase the risk of the recurrence or progression of large and giant PAs. The nomogram model incorporating these risk factors can facilitate prediction of tumor recurrence or progression after transnasal sphenoidal surgery in individual patients.

## Data Availability Statement

The original contributions presented in the study are included in the article/supplementary material. Further inquiries can be directed to the corresponding authors.

## Ethics Statement

The study was reviewed and approved by Ethics Committee of Second Affiliated Hospital, School of Medicine, Zhejiang University.

## Author Contributions

YC and FC performed the analysis and co-wrote the manuscript. AZ, XH, YT, FG, YL, and JC collected the patient information. WY, YW, SC, and XD revised paper. QW and JZ supervised the project, conceived the study, and guided the editing of the manuscript. YC and FC contributed equally to the manuscript. QW and JZ are corresponding authors. All authors contributed to the article and approved the submitted version.

## Conflict of Interest

The authors declare that the research was conducted in the absence of any commercial or financial relationships that could be construed as a potential conflict of interest.

## Publisher’s Note

All claims expressed in this article are solely those of the authors and do not necessarily represent those of their affiliated organizations, or those of the publisher, the editors and the reviewers. Any product that may be evaluated in this article, or claim that may be made by its manufacturer, is not guaranteed or endorsed by the publisher.
